# Demographic, clinical, and functional determinants of antithrombotic treatment in patients with nonvalvular atrial fibrillation

**DOI:** 10.1186/s12872-021-02019-0

**Published:** 2021-08-09

**Authors:** Jose María Mostaza, Carmen Suarez, Jose María Cepeda, Luis Manzano, Demetrio Sánchez, on behalf of the PERFILAR study investigators, Fernando Javier Sánchez Lora, Francisco Ibañez Bermúdez, Ana María Jurado Porcel, Fernando Salgado Ordoñez, Francisco Rivera Civico, Luis Felipe Díez García, Fernando Jaén Águila, Manuel Geraldía Lama, Enrique Peral Gutiérrez-Ceballos, Antonia Domínguez, Francisco Astudillo Martín, Eduardo Aguilar, Juan Ferrando Vela, Alfonso García Aranda, Mercedes Sánchez Cembellín, Juan Francisco López Caleya, Sixto Ruiz, Melchor Rodríguez Gaspar, Alicia Conde Martel, José Luis Hernández Hernández, Ismael Abascal Carrera, Alfonso Pérez del Molino Castellanos, Esther Fernández Pérez, Juan Carlos Martínez Acitores, Luis Miguel Seisdedos Cortes, Laura Abad Manteca, Marco Budiño Sánchez, José Javier Moreno Palomares, Inmaculada Coca Prieto, Ana Isabel Muñoz, Ángel Sánchez Castaño, Lola Ruiz Ribó, Jordi Mascaró, César Morcillo Serra, Teresa Auguet Quintillá, Francesz Marimón, Joaquín Fernández Solá, José María Suriñach, Pablo Marchena, Antoni Riera-Mestre, Pedro Armario, Ferrán García Bragado, Fátima del Molino, Oscar Sacristán, Pere Almagro, Conxita Falgà, Francisco José Muñoz Rodríguez, Riesco, Jorge Romero Requena, José Carlos Arévalo Lorido, Manuela Chiquero Palomo, Ana Isabel de la Cruz, Agustín Pijerro, Elena Fernández Bouza, Juan José González Soler, Manuel Jesús Núñez Fernández, Javier De La Fuente Aguado, José Antonio Díaz Peromingo, Julián Fernández Martín, Rafael Daroca Pérez, Jesús Castiella Herrero, M. Cruz Carreño, Jorge Gómez Cerezo, José Carlos Pontes Navarro, José Felipe Varona Arche, Daniel Ferreiro López, Benjamín Muñoz Calvo, Jesús Manuel Casado Cerrada, María del Pilar Fidalgo Montero, José Manuel Casas Rojo, Benjamín Herreros, Guillermo Cuevas Tascón, Antonio Muiño Miguez, Jorge Marrero Francés, Nicolas Ortega, Javier Trujillo, Julio Sánchez Álvarez, Jose Ignacio Catalán Ramos, Francisco Javier Fresco Benito, Ainhoa Anuzita Alegría, Carlos Teruel, Arturo Artero Mora, Pedro Moral, José Miguel Seguí Ripoll, Fernando Bonilla Rovira, Ana Maestre Peiro

**Affiliations:** 1 Department of Internal Medicine, Hospital Carlos III, Calle Sinesio Delgado, 10, 28029 Madrid, Spain; 2 Department of Internal Medicine, Hospital Universitario de La Princesa, Madrid, Spain; 3 Department of Internal Medicine, Hospital Vega Baja, Orihuela, Alicante, Spain; 4 Department of Internal Medicine, Hospital Ramón Y Cajal, Universidad de Alcalá, Ramón Y Cajal Health Research Institute (IRYCIS), Madrid, Spain; 5 Department of Internal Medicine, Hospital Nuestra Señora De Sonsoles, Ávila, Spain; 6 Hospital Clínico Universitario Virgen de La Victoria, Málaga, Spain; 7 Hospital Comarcal Infanta Margarita, Córdoba, Spain; 8 Complejo Hospitalario Regional Reina Sofía, Córdoba, Spain; 9 Complejo Hospitalario Regional de Málaga (Hospital Carlos Haya), Málaga, Spain; 10 Complejo Hospitalario de Poniente, El Ejido, Almería, Spain; 11 Hospital Torrecárdenas, Almeria, Spain; 12 Hospital Universitario Virgen de Las Nieves, Granada, Spain; 13 Hospital Virgen de Las Montañas, Cádiz, Spain; 14 Complejo Hospitalario Virgen Macarena, Sevilla, Spain; 15 Hospital Quironsalud Sagrado Corazón, Sevilla, Spain; 16 Hospital Infanta Luisa, Sevilla, Spain; 17 Hospital de Alcañiz, Teruel, Spain; 18 Hospital Royo Villanova, Zaragoza, Spain; 19 Hospital Universitario Miguel Servet, Zaragoza, Spain; 20 Hospital San Agustín, Avilés, Asturias Spain; 21 Hospital de Cabueñes, Gijón, Spain; 22 Hospital Comarcal de Inca, Islas Baleares, Spain; 23 Hospital Universitario de Canarias, Santa Cruz de Tenerife, Spain; 24 Hospital Universitario de Gran Canaria Doctor Negrín, Las Palmas de Gran Canaria, Las Palmas, Spain; 25 Hospital Universitario Marqués de Valdecilla, Santander, Spain; 26 Hospital Comarcal de Laredo, Laredo, Spain; 27 Hospital Sierrallana, Torrelavega, Cantabria Spain; 28 Complejo Asistencial Universitario de León, León, Spain; 29 Complejo Asistencial Universitario de Burgos, Burgos, Spain; 30 Complejo Asistencial de Zamora, Zamora, Spain; 31 Hospital Universitario Rio Hortega, Valladolid, Spain; 32 Complejo Asistencial de Ávila, Ávila, Spain; 33 Complejo Asistencial de Segovia, Segovia, Spain; 34 Hospital Santa Bárbara, Ciudad Real, Spain; 35 Hospital Nuestra Señora del Prado, Talavera de La Reina, Toledo, Spain; 36 Hospital Virgen de La Salud, Toledo, Spain; 37 Hospital Virgen de La Luz, Cuenca, Spain; 38 Hospital de La Santa Creu I Sant Pau, Barcelona, Spain; 39 Hospital Cima Sanitas, Barcelona, Spain; 40 Hospital Universitari Joan XXIII, Tarragona, Spain; 41 Hospital Universitari de Sant Joan de Reus, Tarragona, Spain; 42 Hospital Clinic I Provincial, Barcelona, Spain; 43 Hospital Universitari Vall D’Hebron, Barcelona, Spain; 44 Parc Sanitari Sant Joan de Deu, Barcelona, Spain; 45 Hospital Universitari de Bellvitge, Bellvitge Biomedical Research Institute (IDIBELL), L’Hospitalet de Llobregat, Barcelona, Spain; 46 Hospital de Sant Joan Despí Moisés Broggi, Sant Joan Despí, Barcelona, Spain; 47 Hospital Universitari de Girona Dr. Josep Trueta, Girona, Spain; 48 Hospital Quirón Salud del Vallés, Sabadell, Barcelona, Spain; 49 Hospital Santa María, Alcalá de Henares, Madrid, Spain; 50 Hospital Mutua de Terrassa, Terrassa, Barcelona, Spain; 51 Hospital de Mataró, Mataró, Barcelona, Spain; 52 Hospital de Mollet, Mollet del Vallès, Barcelona, Spain; 53 Hospital del Vendrell, El Vendrell, Tarragona, Spain; 54 Hospital Perpetuo Socorro, Badajoz, Spain; 55 Complejo Hospitalario Llerena-Zafra, Llerena, Badajoz, Spain; 56 Complejo Hospitalario de Cáceres, Cáceres, Spain; 57 Hospital Virgen del Puerto, Plasencia, Cáceres, Spain; 58 Hospital Infanta Cristina, Badajoz, Spain; 59 Complexo Hospitalario Universitario Arquitecto Marcide-Novoa Santos, Ferrol, A Coruña, Spain; 60 Complexo Hospitalario Universitario de Ourense, Ourense, Spain; 61 Complexo Hospitalario Universitario de Pontevedra, Pontevedra, Spain; 62 Hospital de Povisa, Vigo, Pontevedra, Spain; 63 Complejo Hospitalario Universitario de Santiago, Santiago de Compostela, A Coruña, Spain; 64 Complejo Hospitalario de Vigo, Vigo, Pontevedra, Spain; 65 Complejo Hospital San Pedro, Logroño, La Rioja Spain; 66 Fundación Hospital Calahorra, Calahorra, La Rioja Spain; 67 Hospital Puerta de Hierro, Madrid, Spain; 68 Hospital Universitario Infanta Sofía, San Sebastián de Los Reyes, Madrid, Spain; 69 Hospital Clínico San Carlos, Madrid, Spain; 70 Hospital Universitario HM Montepríncipe, Boadilla del Monte, Madrid, Spain; 71 Hospital Universitario 12 de octubre, Madrid, Spain; 72 Hospital Universitario Príncipe de Asturias, Alcalá de Henares, Madrid, Spain; 73 Hospital Universitario de Getafe, Getafe, Madrid, Spain; 74 Hospital Universitario del Henares, Coslada, Madrid, Spain; 75 Hospital Universitario Infanta Cristina, Parla, Madrid, Spain; 76 Hospital Universitario Fundación Alcorcón, Alcorcón, Madrid, Spain; 77 Hospital Universitario Infanta Leonor, Madrid, Spain; 78 Hospitalario General Universitario Gregorio Marañón, Madrid, Spain; 79 Hospital de Fuenlabrada, Fuenlabrada, Madrid, Spain; 80 Hospital Virgen de La Arrixaca, El Palmar, Murcia, Spain; 81 Hospital Santa Lucía, Cartagena, Murcia, Spain; 82 Complejo Hospitalario de Navarra, Pamplona, Navarra Spain; 83 Policlínica San José, Vitoria, Álava Spain; 84 Hospital Santa Marina, Bilbao, Vizcaya Spain; 85 Clínica Virgen Blanca, Bilbao, Vizcaya Spain; 86 Hospital Gral. Castellón, Castellón de La Plana, Castellón Spain; 87 Hospital Doctor Peset, Valencia, Spain; 88 Hospital La Fe, Valencia, Spain; 89 Hospital Universitario San Juan, Alicante, Spain; 90 Hospital General de Elche. Elche, Alicante, Spain; 91 Hospital del Vinalopó, Elche, Alicante, Spain

**Keywords:** Antithrombotic treatment, Direct-acting oral anticoagulants (DOACs), Nonvalvular atrial fibrillation (NVAF), Vitamin K antagonists (VKAs)

## Abstract

**Background:**

This study assessed the sociodemographic, functional, and clinical determinants of antithrombotic treatment in patients with nonvalvular atrial fibrillation (NVAF) attended in the internal medicine setting.

**Methods:**

A multicenter, cross-sectional study was conducted in NVAF patients who attended internal medicine departments for either a routine visit (outpatients) or hospitalization (inpatients).

**Results:**

A total of 961 patients were evaluated. Their antithrombotic management included: no treatment (4.7%), vitamin K antagonists (VKAs) (59.6%), direct oral anticoagulants (DOACs) (21.6%), antiplatelets (6.6%), and antiplatelets plus anticoagulants (7.5%). Permanent NVAF and congestive heart failure were associated with preferential use of oral anticoagulation over antiplatelets, while intermediate-to high-mortality risk according to the PROFUND index was associated with a higher likelihood of using antiplatelet therapy instead of oral anticoagulation. Longer disease duration and institutionalization were identified as determinants of VKA use over DOACs. Female gender, higher education, and having suffered a stroke determined a preferential use of DOACs.

**Conclusions:**

This real-world study showed that most elderly NVAF patients received oral anticoagulation, mainly VKAs, while DOACs remained underused. Antiplatelets were still offered to a proportion of patients. Longer duration of NVAF and institutionalization were identified as determinants of VKA use over DOACs. A poor prognosis according to the PROFUND index was identified as a factor preventing the use of oral anticoagulation.

## Background

Atrial fibrillation (AF) is the most common type of sustained cardiac arrhythmia, and its prevalence rises with age, with about 18% of patients older than 80 years being affected [1]. Nonvalvular AF (NVAF) is strongly associated with increased morbidity and mortality related to ischemic stroke and systemic thromboembolism [2, 3]. The risk of stroke in AF patients is about fivefold higher than in the non-AF population [4], and AF-related strokes are generally more severe, with increased risk of death and disability compared to strokes from other causes [5]. Elderly patients with AF are at higher risk of stroke than younger AF patients [6, 7]. Indeed, age ≥ 75 years is a significant risk factor comparable to a history of stroke for the assessment of stroke risk by the CHA_2_DS_2_-VASc score [8]. Prevention of stroke is therefore imperative in AF patients, particularly in elderly patients.

Oral anticoagulation (OAC) with vitamin K antagonists (VKA) has traditionally been the mainstay for stroke prevention in AF based on the robust clinical evidence of their efficacy in preventing stroke or systemic embolism and reducing mortality [9]. However, VKAs have several known limitations, including the risk of major bleeding complications, especially intracranial hemorrhage, many food and drug interactions, and the need for frequent coagulation monitoring due to their narrow therapeutic window. Direct-acting oral anticoagulants (DOACs) targeting thrombin or factor Xa emerged as a welcome addition for stroke prevention in AF. These agents have predictable pharmacodynamic effects, allowing fixed dosing without the need for anticoagulation monitoring [10]. DOACs, such as rivaroxaban, dabigatran, apixaban and edoxaban, have demonstrated to be noninferior to warfarin in stroke prevention without an increased risk of major bleeding [11–15]. Based on their favorable efficacy, safety profile and convenience of use, DOACs are recommended over VKAs for stroke prevention in most patients with NVAF [16].

Adequate selection of antithrombotic therapy for stroke prevention is critical for improving the clinical outcome of patients with NVAF. Several clinical practice guidelines have been developed to guide the management of AF patients, providing clinicians with recommendations on individualization of treatment based on the patient’s characteristics [16, 17]. However, the implementation of guideline recommendations in routine clinical practice may be suboptimal. OAC is still underused in AF patients who are at high risk of stroke, and many patients are instead treated with antiplatelet agents or do not receive antithrombotic treatment [18, 19]. Accordingly, despite the higher risk of stroke and bleeding in elderly NVAF patients [20], anticoagulation has been traditionally underused in this population mainly due to the high frequency of associated comorbidities, including cardiovascular and kidney disease, multiple drug therapy, and concerns about cognitive impairment and risk of falls and bleeding [21].

To date, limited data are available on the clinical management of NVAF patients, particularly in those patients attended in the internal medicine setting, where these patients are typically managed. There is therefore a need to identify current therapies used for stroke prevention and the factors that may guide the selection of treatment in the real-world setting. A better understanding of treatment patterns and factors potentially influencing treatment strategy is crucial to know whether clinical practice is in line with treatment recommendations of the current guidelines*.* Knowledge about the clinical management of patients with NVAF in clinical practice may improve OAC utilization for stroke prevention and outcomes.

Based on this background, we conducted a cross-sectional study to describe the demographic, functional, and clinical characteristics of patients with NVAF attending internal medicine departments in Spanish hospitals and the potential factors associated with antithrombotic treatment patterns.

## Methods

### Study design and patients

A multicenter, cross-sectional observational study was conducted in internal medicine departments from 93 hospitals distributed across Spain.

Eligible patients were consecutive adult patients (aged ≥ 18 years) diagnosed with NVAF (defined as the rhythm disturbance occurring in the absence of rheumatic mitral stenosis or a prosthetic heart valve) who attended the internal medicine departments either for a routine visit (outpatients) or hospitalization (inpatients) for any reason during the 9-month enrolment period. Patients were selected on the basis of the information recorded in the medical charts. The patients must have been diagnosed with NVAF before the study inclusion, and disease-related data (type of NVAF, disease duration, etc.) was collected from medical charts. Patients currently receiving anticoagulant therapy for venous thromboembolism and patients participating in a clinical trial with anticoagulant or antiplatelet agents in the previous six months were excluded.

A cross-sectional chart review was performed to collect patients’ sociodemographic, functional and clinical data, as well as treatment-related data. The social, functional, and cognitive status of patients was also assessed through face-to-face interviews with patients at the time of the study visit (cross-sectional evaluation). Cognitive impairment was evaluated using the Pfeiffer questionnaire [22, 23], and data on functional disability assessed using the Barthel index [24] was collected. Patient comorbidity was measured by using the Charlson comorbidity index [25] (absent of comorbidity: score = 0–1; low comorbidity: score = 2; severe comorbidity: score ≥ 3). The PROFUND index score [26] was calculated to estimate one-year mortality risk as follows: low-risk (12.1–14.6%) for a score of 0–2, low-intermediate risk (21.5–31.5%) for a score of 3–6, intermediate-high risk (45–50%) for a score of 7–10, and high-risk (61.3–68%) for a score ≥ 11. Physician’s estimation of the patient’s life expectancy (< 6 or ≥ 6 months) was also assessed. The risk of ischemic stroke and bleeding was assessed by CHA_2_DS_2_-VASc score [8] and HAS-BLED score [27], respectively.

The independent ethics committee of La Princesa University Hospital (Madrid, Spain) approved the study. All patients gave their written informed consent before their inclusion in the study. The study was carried out in accordance with the Declaration of Helsinki and Good Clinical Practice Guidelines and applicable regulatory requirements.

### Statistical analysis

In order to describe the demographic, functional, and clinical characteristics of patients on each antithrombotic treatment approach, patients included in the study were categorized into five groups according to the antithrombotic therapy used for their clinical management as follows: no treatment, VKAs (acenocumarol or warfarin), DOACs (apixaban, rivaroxaban or dabigatran), antiplatelet drugs (aspirin and/or other antiplatelets), and antiplatelet plus anticoagulant drugs. Only patients who fulfilled the eligibility criteria and could be categorized in any of these treatment groups were considered as evaluable for the study analysis. A descriptive analysis of the characteristics of each treatment group was performed using measures of central tendency and dispersion (mean [± standard deviation], median [interquartile range]) for quantitative variables, and counts and percentages for qualitative variables. The Kolmogorov–Smirnov test and the Shapiro–Wilk test were used for checking normality of data. The Kruskal–Wallis test was used to compare continuous non-parametric variables and the ANOVA to compare continuous parametric variables. The Chi-square or Fisher’s exact test was used for comparison of categorical variables.

Stepwise binary logistic regression analyses were conducted to assess the potential factors associated with the use of specific treatment strategies whose comparison is of clinical relevance. Thus, factors associated with OAC (VKAs, DOACs, or any OAC plus antiplatelet therapy) versus antiplatelet therapy and with VKAs versus DOACs were assessed. Clinically relevant variables and those with a *p* < 0.2 were included in each multivariate model with stepwise selection to determine independent factors associated with each treatment strategy used for clinical management. Odds ratio (OR) and 95% confidence interval (95% CI) were calculated.

All analyses were performed using the Statistical Package for the Social Sciences (SPSS) version 18.0 (SPSS Inc., Chicago, IL, USA).

## Results

### Patients and treatment

**Fig. 1:**
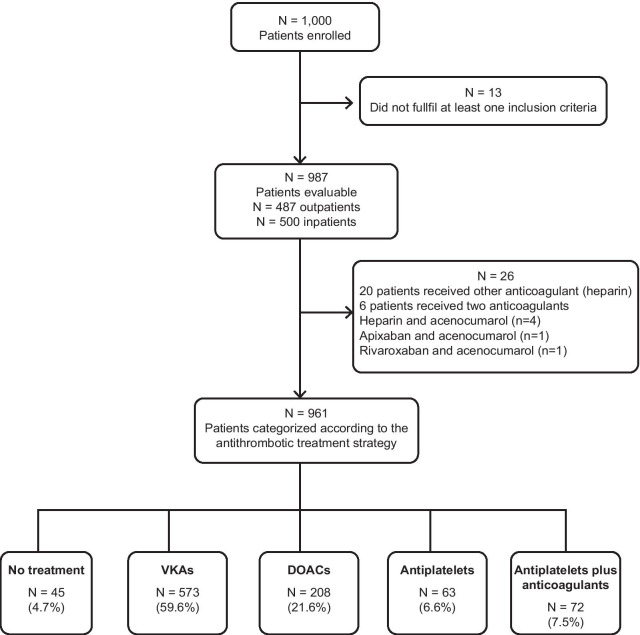
Study patient flow chart

A total of 1000 patients from 93 hospitals distributed across Spain were enrolled in the study from March to October 2015. Thirteen patients were excluded from the analysis, as they did not meet at least one inclusion criteria. Therefore, a total of 987 patients were included in the study analysis. A total of 26 patients could not be categorized in any of the five treatment groups. As a result, 961 patients were evaluable for the study analysis (Fig. 1).

Most patients received antithrombotic treatment (> 95%). Overall, 88.7% of patients received OAC (VKAs, DOACs, or any OAC plus antiplatelet therapy). The majority of patients were treated with VKAs (nearly 60% of patients), and DOACs were received by less than one-quarter of patients. Antiplatelet therapy alone was used in less than 7% of patients, and OAC plus antiplatelet therapy was received by nearly 8% of patients (Fig. 1).

### Demographic and functional characteristics, morbidity and life expectancy

The demographic and functional characteristics, morbidity and life expectancy of patients according to the antithrombotic treatment strategy are shown in Table 1. The median age of patients was 81 years, with similar proportions of male and female patients. Most patients were not institutionalized (> 90%) and had no cognitive impairment according to the Pfeiffer questionnaire (> 60% of patients in all groups). About 65% of patients were not dependent on assistance for activities of daily living (ADLs). The median Charlson comorbidity index score was 7 (high comorbidity). Most patients (90%) had a life expectancy ≥ 6 months according to the investigator’s estimation.

**Table 1:** Patient’s sociodemographic and functional characteristics, morbidity and life expectancy, according to antithrombotic treatment strategy

Characteristics	No treatment	VKAs	DOACs	Antiplatelets	Antiplatelets + anticoagulants	Overall	*p* value
No. (%)	45 (4.7)	573 (59.6)	208 (21.6)	63 (6.6)	72 (7.5)	961 (100.0)	**–**
*Age*, median (IQR), years ^a^	83.0 (74.5–88.0)	81.0 (75.0–86.0)	80.0 (74.0–85.0)	83.0 (76.0–88.0)	78.0 (70.0–82.8)	81.0 (75.0–86.0)	**0.001**
≥ 85 years, n (%)	19 (42.2)	175 (30.5)	56 (26.9)	27 (42.9)	11 (15.3)	288 (30.0)	**0.002**
*Gender*, male, n (%) ^b^	24 (53.3)	285 (49.7)	91 (43.8)	30 (47.6)	46 (63.9)	476 (49.5)	0.059
*BMI*, median (IQR), Kg/m^2 a^	24.6 (22.7–27.7)	28.0 (24.9–31.7)	27.8 (24.2–31.4)	26.4 (23.5–30.7)	29.2 (25.3–33.6)	27.7 (24.8–31.6)	**0.001**
*Educational* level, n (%) ^b^							**0.047**
Did not complete compulsory education	10 (23.8)	114 (20.9)	36 (17.9)	10 (16.9)	14 (20.0)	184 (20.0)	
Primary education	22 (52.4)	348 (63.7)	113 (56.2)	33 (55.9)	45 (64.3)	561 (61.1)	
Secondary education	4 (9.5)	61 (11.2)	33 (16.4)	10 (16.9)	8 (11.4)	116 (12.6)	
University or higher	6 (14.3)	23 (4.2)	19 (9.5)	6 (10.2)	3 (4.3)	57 (6.2)	
*Institutionalized*, n (%) ^c^	4 (8.9)	41 (7.2)	2 (1.0)	9 (14.3)	2 (2.8)	58 (6.1)	** < 0.001**
*Dependence in * *ADLs, n (%) *^*b*^	18 (40.9)	187 (32.7)	63 (30.3)	34 (54.0)	25 (34.7)	327 (34.1)	**0.008**
*Barthel index score* < 60 (severe/total dependence), n (%) ^b^	15 (33.3)	84 (14.7)	33 (15.9)	23 (36.5)	7 (9.7)	162 (16.9)	** < 0.001**
*Charlson index, median (IQR) *^*c*^	7.0 (5.5–9.0)	7.04 (6.0–9.0)	7.0 (5.0–8.0)	8.0 (6.0–9.0)	8.0 (6.0–10.0)	7.04 (6.0–9.0)	**0.009**
*Cognitive impairment (SPMSQ score), n (%) *^*b*^	13 (28.9)	172 (30.2)	65 (31.3)	23 (36.5)	18 (25.0)	291 (30.4)	0.692
Score > 7 (severe)	3 (6.7)	86 (15.0)	37 (17.8)	12 (19.0)	7 (9.7)	145 (15.1)	0.194
*PROFUND index score, median (IQR)*^*a*^	6.0 (1.0–10.5)	3.0 (0.0–6.0)	3.0 (0.0–5.8)	5.0 (0.0–10.0)	2.0 (0.0–5.0)	3.0 (0.0–6.0)	** < 0.001**
*1-year* mortality risk, n (%) ^b^							** < 0.001**
Low (12.1–14.6%)	14 (31.1)	258 (45.0)	99 (47.6)	17 (27.0)	37 (51.4)	425 (44.2)	
Low-intermediate (21.5–31.5%)	10 (22.2)	191 (33.3)	72 (34.6)	17 (27.0)	24 (33.3)	314 (32.7)	
Intermediate-high (45.0–50.0%)	10 (22.2)	72 (12.6)	19 (9.1)	17 (27.0)	9 (12.5)	127 (13.2)	
High (61.3–68.0%)	11 (24.4)	52 (9.1)	18 (8.7)	12 (19.0)	2 (2.8)	95 (9.9)	
*Life* expectancy, n (%) ^b^							** < 0.001**
≥ 6 months	34 (75.6)	522 (91.1)	196 (94.2)	53 (84.1)	68 (94.4)	873 (90.8)	
< 6 months	11 (24.4)	51 (8.9)	12 (5.8)	10 (15.9)	4 (5.6)	88 (9.2)	

*ADL* activities of daily living, *BMI* body mass index, *DOAC* direct-acting oral anticoagulant, *IQR* interquartile range, *SD* standard deviation, *SPMSQ* Pfeiffer Short Portable Mental Status Questionnaire, *VKA* vitamin K antagonist

For normally distributed data, mean and standard deviation are used, and for data not normally distributed, median with interquartile range are used. ^a^Non-parametric Kruskal–Wallis test, ^b^Chi-square test, ^c^Fisher’s exact test

Compared with autonomous patients, non-autonomous patients were older (84 [80–88] vs. 78 [72–83] years; *p* < 0.001), predominantly female (62.3% vs. 43.7%; *p* < 0.001), and with higher risk of stroke and bleeding according to CHA_2_DS_2_-VASc (5.6 ± 1.5 vs. 4.4 ± 1.5; *p* < 0.001) and HAS-BLED (3.4 ± 1.1 vs. 2.8 ± 1.1; *p* < 0.001) scores. Similarly, patients with cognitive impairment had a more advanced age (83 [79–88] vs. 79.0 [73–84] years; *p* < 0.001), were mostly women (61.2% vs. 45.1%; *p* < 0.001), and had higher CHA_2_DS_2_-VASc (5.2 ± 1.6 vs. 4.6 ± 1.6; *p* < 0.001) and HAS-BLED (3.1 ± 1.2 vs. 2.9 ± 1.1; *p* < 0.001) scores compared with patients without cognitive impairment. Additionally, the median age of patients with < 4 comorbidities was 64 (54–67) years while it was 81 (75–86) years in patients with ≥ 4 comorbidities (*p* < 0.001) in whom CHA_2_DS_2_-VASc and HAS-BLED scores were higher (vs. < 4 comorbidities) (CHA_2_DS_2_-VASc: 4.9 ± 1.5 vs. 1.9 ± 1.0; *p* < 0.001; HAS-BLED: 3.1 ± 1.1 vs. 1.2 ± 1.0; *p* < 0.001). Patients with a life expectancy < 6 months (vs. ≥ 6 months) were older (86 [81–91] vs. 80 [74–85] years; *p* < 0.001) and with higher CHA_2_DS_2_-VASc (5.3 ± 1.6 vs. 4.7 ± 1.6; *p* < 0.001) and HAS-BLED (3.5 ± 1.2 vs. 2.9 ± 1.1; *p* < 0.001) scores.

The differences in sociodemographic and functional characteristics, morbidity and life expectancy between the patients undergoing different antithrombotic treatment strategies are shown in Table 1. There were statistically significant differences in terms of age and educational level between the different treatment groups. Significant differences in functional characteristics (institutionalization, dependence in ADLs, and functional disability measured by the Barthel index) were also observed between the different treatment groups except in terms of cognitive impairment. In addition, there were statistically significant differences in estimated prognostic characteristics, including comorbidity (Charlson comorbidity index score), 1-year mortality risk (PROFUND index) and life expectancy (investigator’s clinical judgment) among the patients treated with the different antithrombotic treatment strategies.

### Clinical characteristics

Clinical characteristics according to the antithrombotic treatment strategy are shown in Table 2. Most patients were diagnosed with permanent NVAF (70%), with a median time since diagnosis of over four years. The most common comorbidities were hypertension (90%), congestive heart failure (CHF) (54%), renal disease (43%), and diabetes mellitus (40%). A history of bleeding was present in less than 15% of patients.

**Table 2:** Clinical characteristics of the overall population and according to antithrombotic treatment strategy

Characteristics	No treatment	VKAs	DOACs	Antiplatelets	Antiplatelets + anticoagulants	Overall	*p* value
No. (%)	45 (4.7)	573 (59.6)	208 (21.6)	63 (6.6)	72 (7.5)	961 (100.0)	–-
*Inpatient/Outpatient,* n (%)	21 (46.7)/ 24 (53.3)	301(52.5)/ 272 (47.5)	70 (33.7)/ 138 (66.3)	46 (73.0)/ 17 (27.0)	43 (59.7)/ 29 (40.3)	481(50.1)/ 480 (49.9)	–-
*Type of NVAF*, n (%) ^b^							** < 0.001**
Paroxysmal	21 (46.7)	90 (15.7)	52 (25.0)	26 (41.3)	14 (19.4)	203 (21.1)	
Persistent	6 (13.3)	47 (8.2)	19 (9.1)	6 (9.5)	7 (9.7)	85 (8.8)	
Permanent	18 (40.0)	436 (76.1)	137 (65.9)	31 (49.2)	51 (70.8)	673 (70.0)	
*Time since diagnosis*, median (IQR), years ^a^	2.5 (0.1–7.2)	4.7 (2.3–9.0)	3.0 (1.3–8.0)	5.0 (1.0–8.2)	3.9 (1.4–8.3)	4.2 (1.7–8.6)	** < 0.001**
*Comorbidities*, n (%) ^b^							
Active neoplasia	11 (24.4)	76 (13.3)	24 (11.5)	12 (19.0)	10 (13.9)	133 (13.8)	0.149
COPD	9 (20.0)	164 (28.6)	53 (25.5)	15 (23.8)	21 (29.2)	262 (27.3)	0.631
Renal disease/CKD	17 (37.8)	180 (31.4)	54 (26.0)	23 (36.5)	24 (33.3)	298 (31.0)	0.336
*Cardiovascular* history, n (%)							
Congestive heart failure	21 (46.7)	323 (56.4)	106 (51.0)	28 (44.4)	44 (61.1)	522 (54.3)	0.147
Ischaemic disease ^d^	1 (2.2)	101 (17.6)	28 (13.5)	12 (19.0)	37 (51.4)	179 (18.6)	** < 0.001**
Peripheral artery disease	1 (2.2)	43 (7.5)	17 (8.2)	9 (14.3)	18 (25.0)	88 (9.2)	** < 0.001**
Stroke/TIA	8 (17.8)	95 (16.6)	47 (22.6)	17 (27.0)	22 (30.6)	189 (19.7)	**0.016**
Arterial thromboembolism/venous thromboembolic disease, n (%)	2 (4.4)	29 (5.1)	9 (4.3)	2 (3.2)	2 (2.8)	44 (4.6)	0.885
Hypertension, n (%)	35 (77.8)	519 (90.6)	191 (91.8)	54 (85.7)	65 (90.3)	864 (89.9)	**0.047**
Diabetes mellitus, n (%)	10 (22.2)	237 (41.4)	72 (34.6)	31 (49.2)	36 (50.0)	386 (40.2)	**0.007**
Abnormal hepatic function ^e^, n (%)	2 (4.4)	37 (6.5)	14 (6.7)	6 (9.5)	2 (2.8)	61 (6.3)	0.590
Abnormal renal function ^f^, n (%)	20 (44.4)	249 (43.5)	82 (39.4)	31 (49.2)	32 (44.4)	414 (43.1)	0.695
*Prior bleeding*, n (%) ^b^	8 (17.8)	71 (12.4)	33 (15.9)	9 (14.3)	10 (13.9)	131 (13.6)	0.678
*CHA*_*2*_*DS*_*2*_*-VASc score*, median (IQR) ^a^	4.0 (4.0–5.5)	5.0 (4.0–6.0)	5.0 (4.0–6.0)	5.0 (4.0–6.0)	5.0 (4.0–6.8)	5.0 (4.0–6.0)	0.097
*Risk* categories, n (%) ^c^							** < 0.001**
0	4 (8.9)	0 (0.0)	0 (0.0)	1 (1.6)	0 (0.0)	5 (0.5)	
1	0 (0.0)	6 (1.0)	1 (0.5)	0 (0.0)	1 (1.4)	8 (0.8)	
≥ 2	41 (91.1)	567 (99.0)	207 (99.5)	62 (98.4)	71 (98.6)	948 (98.6)	
Score ≥ 5, n (%)	19 (46.4)	330 (58.2)	112 (54.6)	39 (62.9)	47 (66.0)	547 (57.6)	
*HAS-BLED score*, median (IQR) ^a^	3.0 (2.0–3.0)	3.0 (2.0–4.0)	3.0 (2.0–4.0)	4.0 (3.0–4.0)	4.0 (3.0–5.0)	3.0 (2.0–4.0)	** < 0.001**
Risk categories, n (%) ^b^							** < 0.001**
< 3	22 (48.9)	228 (39.8)	75 (36.1)	7 (11.1)	5 (6.9)	337 (35.1)	
≥ 3	23 (51.1)	345 (60.2)	133 (63.9)	56 (88.9)	67 (93.1)	624 (64.9)	

*NVAF* non-valvular atrial fibrillation, *COPD* chronic obstructive pulmonary disease, *CKD* chronic kidney disease, *DOAC* direct-acting oral anticoagulant, *IQR* interquartile range, *SD* standard deviation, *TIA* transient ischemic attack, *VKA* vitamin K antagonist

For normally distributed data, mean and standard deviation are used, and for data not normally distributed, median with interquartile range are used. ^a^Non-parametric Kruskal–Wallis test, ^b^Chi-square test, ^c^Fisher’s exact test. ^d^Includes myocardial infarction and stable coronary artery disease, ^e^chronic hepatic disease (e.g. cirrhosis) or biochemical data indicative of significant hepatic damage (e.g. bilirubin > 2 × the upper normal limit, associated with AST/ALT > 3 × the upper normal limit, etc.), ^f^chronic dialysis, renal transplant or serum creatinine ≥ 200 μmol/l) (yes/no) and renal disease staging based on the glomerular filtration rate (GFR) according to the Kidney Disease Improving Global Outcomes (KDIGO) guidelines G3a, 45–59 mL/min/1,73 m^2^; G3b, 30–44 mL/min/1.73 m^2^; G4, 15–29 mL/min/1.73 m^2^; or G5, < 15 mL/min/1.73 m^2^

The vast majority of patients had high thromboembolic risk according to the CHA_2_DS_2_-VASc score (≥ 2), with more than 45% of patients with a score ≥ 5 in all treatment groups. Most patients (> 60%) had a HAS-BLED score ≥ 3.

The differences in clinical characteristics between the patients undergoing different antithrombotic treatment strategies are shown in Table 2. There were statistically significant differences in the type of NVAF and the median time since diagnosis among treatment groups. Cardiovascular history also differed among the patients treated with each antithrombotic therapy except for CHF. No significant differences were found in the percentage of patients with high thromboembolic risk (CHA_2_DS_2_-VASc ≥ 2) who were treated with each antithrombotic therapy. However, the percentage of patients with high hemorrhagic risk (HAS-BLED) differed significantly by treatment strategy (*p* < 0.001).

### Factors associated with antithrombotic treatment patterns

The results of the multivariate analysis performed to identify the independent factors associated with the use of OAC versus antiplatelet therapy and of DOACs versus VKAs are summarized in Table 3.

**Table 3:** Factors associated with treatment strategy for NVAF by multivariate analysis

Endpoint^a^	OR (95% CI)		*p* value
*Anticoagulants versus antiplatelet therapy*^b^			
*PROFUND Index (1-year mortality risk)* (Referral category: low)			** < 0.0001**
Low-intermediate	0.455 (0.221–0.938)		**0.033**
Intermediate-high	0.144 (0.067–0.312)		** < 0.0001**
High	0.133 (0.056–0.316)		** < 0.0001**
*Type of NVAF* (Referral category: paroxysmal)			** < 0.0001**
Persistent	2.077 (0.796–5.422)		0.135
Permanent	4.122 (2.281–7.450)		** < 0.0001**
*Congestive heart failure* (yes vs. no)	2.084 (1.173–3.703)		**0.012**
*VKAs versus DOACs*^c^			
*Gender* (male vs. female)	0.677 (0.478–0.959)		**0.028**
*Educational level* (Referral category: did not complete compulsory education)			**0.004**
Primary education	0.959 (0.617–1.491)		0.853
Secondary education	0.534 (0.297–0.961)		**0.036**
University or higher	0.337 (0.159–0.715)		**0.023**
*Institutionalization* (yes vs. no)	7.744 (1.816–33.027)		**0.006**
*Time since diagnosis of NVAF*	1.045 (1.011–1.080)		**0.009**
*Prior stroke/TIA* (yes vs. no)	0.591 (0.386–0.903)		**0.015**

CI: confidence interval, DOAC: direct-acting oral anticoagulant, NVAF non-valvular atrial fibrillation, OR: odds ratio, TIA: transient ischemic attack, VKA: vitamin K antagonist

^a^The covariates included in the univariate models were as follows: age, gender, educational level, institutionalization, dependence in activities of daily living (ADLs), PROFUND index, Charlson comorbidity index, cognitive impairment (SPMSQ score), life expectancy (physician’s criteria), type of NAVF, time since diagnosis of NVAF, active neoplasia, dementia, diabetes mellitus, hypertension, congestive heart failure (CHF), ischaemic disease (myocardial infarction, and/or stable coronary arterial disease), peripheral artery disease (PAD), cerebrovascular disease (prior stroke/transient ischemic attack [TIA]), venous thromboembolic disease, prior bleeding, predisposition to bleeding, abnormal hepatic function, abnormal renal function, and thromboembolic risk (CHA_2_DS_2_-VASc score)

^b^The variables included in the multivariate analysis were institutionalization (*p* = 0.005), dependence in ADLs (*p* = 0.001), 1-year risk mortality risk (PROFUND index) (*p* < 0.0001), Charlson comorbidity index (*p* = 0.012), life expectancy (*p* =0.031 ), type of NVAF (*p* < 0.001), and dementia (*p* = 0.007). Age (*p* =0.098), diabetes mellitus (*p* = 0.174), arterial hypertension (*p* =0.183), CHF (*p* = 0.092), PAD (*p* = 0.183), and prior stroke/TIA (*p* = 0.138) were also considered in the multivariate model (*p* < 0.2)

^c^The variables included in the multivariate analysis were educational level (*p* = 0.008), institutionalization (*p* = 0.005), Charlson comorbidity index (*p* = 0.029), type of NVAF (*p* = 0.009), and time since diagnosis of NVAF (*p* < 0.008). Gender (*p* = 0.139), life expectancy (*p* = 0.159), diabetes mellitus (*p* = 0.089), CHF (*p* = 0.180), ischemic disease (*p* = 0.167), and prior stroke/TIA (*p* = 0.055) were also considered in the multivariate model (*p* < 0.2). Prior bleeding (*p* = 0.208) and abnormal renal function (0.314) were also included as relevant determinants of anticoagulant treatment

#### Factors associated with the use of OAC versus antiplatelet therapy

The presence of CHF (OR, 2.084) and permanent NVAF (OR, 4.122) was associated with preferential use of OAC over antiplatelets, while intermediate- to high-mortality risk according to the PROFUND index (vs. low mortality risk) (OR, 0.455; 0.144, and 0.133 respectively) was associated with a higher likelihood of using antiplatelet therapy alone instead of OAC.

#### Factors associated with the use of DOACs versus VKAs

Factors significantly associated with the use of VKAs over DOACs were longer time since diagnosis (OR, 1.045) and institutionalization (OR, 7.744). However, female gender (vs. male), secondary or university or higher studies, and having suffered a stroke/transient ischemic attack (OR, 0.591) were factors associated with preferential use of DOACs.

## Discussion

The present study assessed the demographic, functional, and clinical characteristics associated with antithrombotic treatment patterns in a large real-life population of patients with NVAF. The median age of patients was about 80 years, and most patients had permanent NVAF, with a median disease duration of over four years. As expected, this elderly population had a high comorbidity burden, particularly cardiovascular risk factors and disease, a very high thromboembolic risk and a moderate to high risk of bleeding. Most patients were autonomous and had no cognitive impairment. Patients with worse functional status and worse prognosis in terms of survival were those with more advanced age and with a higher risk of stroke and bleeding.

Despite being a population comprised of elderly patients with a high comorbidity burden, we found that the vast majority of patients (95%) were receiving antithrombotic treatment due to their high thromboembolic risk. Most patients received VKA treatment (60%) while DOACs were given in less than one-quarter of patients. Of note, antiplatelet therapy alone was prescribed in less than 7% of patients with NVAF in routine clinical practice.

Older age is a common reason given for not prescribing anticoagulation [20, 28]. Thus, the high use of OAC shown in this real-world analysis (88%) was especially interesting, since 75% of patients were older than 75 years and 30% of patients were aged ≥ 85 years. This study has therefore highlighted that age alone was not considered as a contraindication for anticoagulation. The underuse of OAC in elderly patients is mainly due to overestimation of the risk of bleeding in these patients [29]. However, the benefit of stroke prevention outweighs the potential increased risk of bleeding in these patients [30, 31], in whom a higher net clinical benefit has been demonstrated compared to the younger population due to their higher thromboembolic risk [32]. We found a substantial increase in the use of OAC in elderly patients compared to prior studies conducted in octogenarian AF patients in Spain, where only half of the patients received anticoagulation [33]. This difference may suggest changes in physicians’ perceptions of the benefits and risks of OAC for older NVAF patients based on the available evidence demonstrating its benefit in this population [20, 34].

Of note, the percentage of patients who received no antithrombotic treatment in our series (4.7%) was similar to that previously reported in the European population of the GLORIA-AF registry (4.1%) [35]. However, compared to other European studies, our analysis showed a significantly lower proportion of untreated patients [36, 37]. A higher rate of untreated patients has also been reported among elderly patients in the internal medicine setting in Spain (13.8%) [38].

Although a history of prior bleeding has traditionally been associated with anticoagulation under-prescription in elderly patients [39], bleeding history was not identified as a factor contributing to anticoagulation underuse in our study. Indeed, a high HAS-BLED score should not be a reason for non-prescription of anticoagulation but rather a tool to identify and control modifiable bleeding risk factors to reduce the risk of bleeding [40, 41].

Antiplatelet therapy has traditionally been used instead of anticoagulation to prevent the risk of bleeding despite the demonstrated greater efficacy of anticoagulant treatment over antiplatelet agents without increasing major bleeding [42]. Nevertheless, we found that the use of antiplatelet therapy alone (7%) was notably lower than that previously reported in primary care (20%) [43]. Interestingly, antiplatelet therapy users were less frequent compared to prior national studies in elderly inpatients (about 18%) [38, 44]. Indeed, we found that compared to outpatients, the proportion of hospitalized patients receiving antiplatelet therapy was higher, probably because these patients are more likely to have frailty and more comorbidity compared to outpatients and thus may not be considered optimal candidates for anticoagulation [28].

Of note, institutionalized patients and those with total or partial dependence for daily activities, who were older and with higher thromboembolic and bleeding risk, were primarily treated with antiplatelets, which were also preferentially used in patients with functional disability and worse prognosis. These findings, therefore, suggest that there might be functional and prognostic factors driving the selection of antiplatelet therapy alone over OAC. Indeed, the univariate analysis showed that institutionalization, dependence for daily activities, 1-year mortality risk assessed by the PROFUND index, and life expectancy (physician’s clinical judgment) were factors significantly associated with preferential use of antiplatelet therapy over OAC. However, among these factors, only 1-year mortality risk was retained in the multivariate model, which showed that poor prognosis (intermediate or high risk) was a factor contributing to OAC non-prescription in favor of antiplatelet therapy alone.

This real-world assessment has also highlighted that the type of NVAF appears to be a factor that has played an essential role in the underuse of OAC in NVAF. In this regard, we found that patients with paroxysmal NVAF were more likely to receive antiplatelet therapy alone instead of OAC compared to patients with permanent NVAF in line with prior reports [1, 35, 45]. The underuse of OAC in favor of antiplatelet therapy in patients with paroxysmal NVAF could be due to the perception of a lower thromboembolic risk associated with this type of NVAF [46]. However, contrary findings regarding its impact on thromboembolic risk have been reported [47, 48]. Nevertheless, according to current guidelines [16], the decision concerning antithrombotic therapy should be based on the patient’s risk of stroke and bleeding, regardless of the type of NVAF.

Regarding the type of OAC, we found that a significant proportion of patients still received VKAs over DOACs despite guideline recommendations [16]. The use of DOACs among patients receiving OAC (24.4%) was similar to that reported in Spanish studies conducted at the regional level during 2015 (24%) and 2011–2014 (25%) [49] and the nationwide FANTASIIA registry (22%) [50]. Thus, a substantial proportion of Spanish NVAF patients who could benefit from DOACs did not receive them, as recognized by experts [51]. In addition, the preference for VKAs over DOACs may be due to the lack of available targeted DOAC reversal agents for patients with bleeding at the time of the study. Moreover, the prescription of DOACs can be challenging in Spain due to the restrictions for their use stated in the national Therapeutic Positioning Report (TPR) [52] and the different administrative requirements of the Spanish Autonomous Communities. This study also found that the use of DOACs in Spain is still far behind other European countries, where an overall rate of DOAC prescription of approximately 50% [35] has been reported. However, geographical differences have been found in prescription patterns [36, 53, 54].

We found that neither the functional characteristics nor the prognosis (PROFUND index) or morbidity were associated with the use of DOACs. Nevertheless, preferential use of VKAs over DOACs was found among institutionalized patients, probably due to a more controlled environment which may enhance VKA treatment adherence and might avoid unacceptable risks derived from poor anticoagulation control. There appear to be sociodemographic factors driving DOAC selection, such as gender and educational level. Thus, educational level was identified as an independent factor associated with the use of DOACs, with a preference of these agents over VKAs in more educated patients, probably also with a higher income, in line with prior reports [55]. This may be explained by better access of patients with a high educational level to the available information on new oral anticoagulants, which may influence treatment decision for stroke prevention. In addition, patients with longer disease duration were also more likely to receive VKA therapy over DOACs probably because prescription of DOACs may have only increased over the past few years due to current guidelines recommendation [16]. A prior history of stroke was also associated with a higher likelihood of receiving DOACs according to the European recommendations [16].

DOAC prescription has been traditionally limited by renal function, especially in elderly patients whose renal function is often unstable and affected by concomitant comorbidities and hospitalizations [56, 57]. However, abnormal renal function was not identified as a factor contributing to DOAC non-prescription, in contrast to other reports in the primary care setting [49]. Indeed, renal impairment should not be a limitation to DOAC prescription and reduced dose regimens of DOACs are approved for use in moderate and severe chronic kidney disease.

The main limitations of this study are due to the research design. This was a cross-sectional study which included a mixed incident and prevalent NVAF population; new starters and switchers from antithrombotic therapy, who were selected from a large sample of hospitals in Spain. However, it is noteworthy that this study did not aim to focus on treatment patterns, such as switching or discontinuation. In addition, we must take into account the obvious limitations of a retrospective chart review that uses patients’ data already recorded in the medical charts for reasons other than research, including incomplete or unrecorded information. A further limitation that should be acknowledged is the unequal number of patients in each group defined by the therapeutic strategy used for NVAF management. However, we should note that these groups were created according to clinical practice, given that patients could be receiving any treatment for NVAF when they were included in the study. Indeed, this study did not aim to assess the differences between these groups, and the comparisons made between them are exploratory and descriptive. Despite these limitations, this study provides valuable real-world data on the profile and treatment patterns of NVAF patients and grounds for discussion on whether antithrombotic treatment strategies are in line with guideline recommendations. Thus, the findings obtained in this analysis may be used to identify critical issues that should be improved in the management of NVAF patients, which might optimize patient care and outcomes. The strengths of this study include a highly representative population of non-selected elderly patients with NVAF in clinical practice, including inpatients and outpatients, attended in the internal medicine departments of more than 90 hospitals distributed homogeneously throughout the country. To our knowledge, this is the first and the most extensive study to assess the real-world characteristics of NVAF patients according to antithrombotic treatment after approval of DOACs in the internal medicine setting in Spain.

## Conclusion

This study showed that the vast majority of elderly patients with NVAF, with high comorbidity burden and high thromboembolic risk, received OAC in the real-world setting, with VKAs as the most frequently prescribed treatment, while DOACs remained underused. Longer duration of AF and institutionalization were identified as determinants of VKA use over DOACs. Antiplatelet therapy was still offered to a proportion of patients. A poor prognosis according to the PROFUND index was identified as a factor preventing the use of OAC.

## Data Availability

The datasets used and/or analyzed during the current study are available from the corresponding author on reasonable request.

## Abbreviations

ADL: Activities of daily living
AF: Atrial fibrillation
CHF: Congestive heart failure
CI: Confidence interval
DOAC: Direct oral anticoagulants
NVAF: Non-valvular atrial fibrillation
OAC: Oral anticoagulation
OR: Odds ratio
VKA: Vitamin K antagonist
